# Role of Yme1 in mitochondrial protein homeostasis: from regulation of protein import, OXPHOS function to lipid synthesis and mitochondrial dynamics

**DOI:** 10.1042/BST20240450

**Published:** 2024-06-12

**Authors:** Kwan Ting Kan, Joel Wilcock, Hui Lu

**Affiliations:** School of Biological Sciences, Faculty of Biology, Medicine and Health, Manchester Academic Health Science Centre, The University of Manchester, Manchester M13 9PT, U.K.

**Keywords:** i-AAA proteinase, mitochondrial protein homeostasis, mitochondrial protein import, oxidative phosphorylation complex, protein function

## Abstract

Mitochondria are essential organelles of eukaryotic cells and thus mitochondrial proteome is under constant quality control and remodelling. Yme1 is a multi-functional protein and subunit of the homo-hexametric complex i-AAA proteinase. Yme1 plays vital roles in the regulation of mitochondrial protein homeostasis and mitochondrial plasticity, ranging from substrate degradation to the regulation of protein functions involved in mitochondrial protein biosynthesis, energy production, mitochondrial dynamics, and lipid biosynthesis and signalling. In this mini review, we focus on discussing the current understanding of the roles of Yme1 in mitochondrial protein import via TIM22 and TIM23 pathways, oxidative phosphorylation complex function, as well as mitochondrial lipid biosynthesis and signalling, as well as a brief discussion of the role of Yme1 in modulating mitochondrial dynamics.

## Introduction

Mitochondria are essential organelles within eukaryotic cells. They not only are the major sites of energy production by carrying out oxidative phosphorylation (OXPHOS), but also play important roles in metabolic and signalling pathways essential for cellular homeostasis [1,2]. Thus, mitochondrial biogenesis and function are highly regulated, and mitochondrial proteostasis is under constant monitoring and remodelling in response to the ever-changing demand of the cells.

Mitochondria are double-membraned organelles of eukaryotic cells, consisting of the mitochondrial outer membrane (MOM), intermembrane space (IMS), mitochondrial inner membrane (MIM), and matrix. The mitochondrial proteome consists of about 1000–1500 proteins [3–6]. Apart from 8 in yeast *Saccharomyces cerevisiae*, and 13 in human, about 99% of all the mitochondrial proteins are nucleus-encoded [6,7], and their biogenesis involves the import of the cytosolic precursor proteins to mitochondrial sub-compartments and subsequent folding and assembly [8]. On the other hand, proteolytic removal of damaged or misfolded and unwanted proteins is a major strategy for mitochondrial protein quality control [9,10]. As such, a network of proteolytic systems located in different mitochondrial sub-compartments is responsible for mitochondrial protein quality control and maintaining mitochondrial proteostasis [9]. Proteases of the AAA (ATPase associated with various cellular activities) superfamily serve as one of the critical components for protein degradation in mitochondria [11]. The MIM hosts two such AAA proteases, i-AAA and m-AAA, which have their catalytic domain exposed to the IMS (i-AAA) and the matrix (m-AAA), respectively [12].

Yme1 (yeast mitochondrial escape 1) forms the hexametric complex i-AAA, which is the only ATP-dependent protease located in the mitochondrial IMS. Cryo-EM structure of a soluble yeast Yme1 revealed a hexametric ring structure with an asymmetric spiral staircase-like conformation, and the allosteric mechanism linking ATP hydrolysis to substrate translocation in Yme1 [13]. Increasing evidence shows that Yme1 (or human homologue YME1L) is a multi-functional protein involved in not only mitochondrial protein turnover, but also protein import, folding, and maturation. As a quality control protease, Yme1 degrades damaged, misfolded, and unwanted substrate proteins, whereas as a regulatory enzyme, it processes or cleaves proteins partially to reveal their masked activities [14–17]. Overall, Yme1 (i-AAA) plays vital roles in the homeostasis of mitochondrial proteins at various levels, from the degradation of proteins at an individual substrate level to the regulation of mitochondrial protein homeostasis at an organelle level, via mediating mitochondrial protein import, mitophagy, fission, and fusion [10,18]. Indeed, our recent mitochondrial proteomic analyses have shown that the abundances of many mitochondrial proteins, found in differential sub-compartments and involved in multitudes of mitochondrial processes, are altered by the absence of Yme1 [19]. The role of Yme1/YME1L on the regulation of mitochondrial dynamics has been well reviewed in detail recently [20]. Here, in this mini review, we will focus more on the current understanding of the role of i-AAA protease Yme1/YME1L in mitochondrial protein import, the function of OXPHOS complexes, and mitochondrial lipid biogenesis and signalling.

### Yme1/YME1L and mitochondrial protein biogenesis via TIM22 and TIM23 translocases

**Figure 1.**
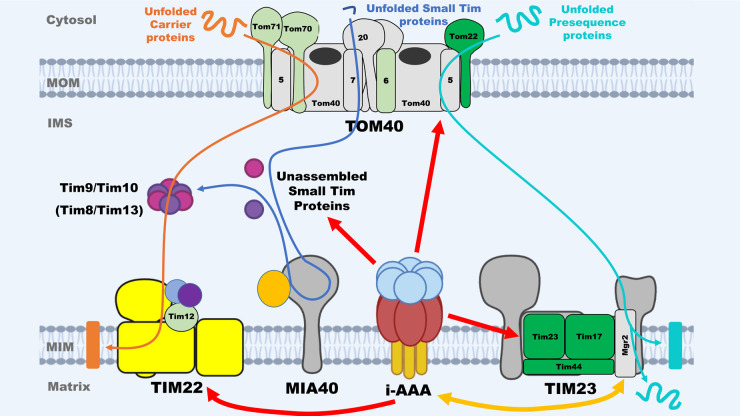
Schematic diagram of the interplay between the i-AAA and mitochondrial protein import pathways based on using yeast *S. cerevisiae* as a model. Here, only the relevant subunits of TOM40, TIM22, TIM23, and MIA40 import complexes are shown. Thick red arrows indicate that i-AAA proteinase proteolytically degrades given substrate proteins, such as the unassembled small Tim proteins. For the subunits of TOM40, TIM22, and TIM23 complexes, green represents confirmed substrates of Yme1, light green represents putative substrates of Yme1, and grey represents no evidence for the proteins to be substrates of Yme1. Stability of TIM22 complex (yellow) is decreased in *Δyme1* yeast cells. In addition, double orange arrow represents the interplay between human TIM23 and YME1L, where human ROMO1 (Mgr2 homologue) is a substrate of YME1L and also mediates the import of YME1L via TIM23 pathway.

Most mitochondrial proteins are imported from the cytosol through the entry gate of the TOM complex, after that TIM22 and TIM23 are two major complexes responsible for continuing the import of MIM and matrix proteins, respectively (Figure 1) [21,22]. About 60% of mitochondrial proteins containing the N-terminal pre-sequence are imported into the matrix or the MIM via the TIM23 pre-sequence pathway in the inner membrane. On the other hand, the hydrophobic MIM proteins without pre-sequences are imported into the inner membrane via the TIM22 translocase with the help of the small Tim proteins in the IMS. Studies have shown that Yme1 plays a role in the quality control and regulation of proteins involved in both the TIM22 and TIM23 import pathways.

A study with human cells under stress conditions identified the TIM23 complex subunit Tim17A as a degradation substrate of YME1L under stress conditions [23], suggesting YME1L played a regulation role in the stress response mechanism to maintain mitochondrial proteostasis via reduced mitochondrial protein import [23]. Similarly, a study using mouse embryonic fibroblast cells under hypoxia showed down-regulation of protein translocase subunits including TIM23 complex subunit Timm17a and Timm23 in WT but not in *Yme1l^−/−^* cells, hence further highlights YME1L role in regulating mitochondrial protein import [24]. Consistently, our recent yeast mitochondrial proteomic analysis identified TIM23 complex subunits, Tim17, Tim23, and Tim44, to be significantly increased in the *YME1* deleted mitochondria and thus putative substrates of Yme1 [19]. Furthermore, YME1L itself was shown to be imported into MIM using the TIM23 translocase that contains the ROMO1 subunit [25]. Whilst ROMO1 is dispensable for general protein import via the TIM23 pathway, it is specifically required for the import of YME1L, and severely reduced levels of YME1L in ROMO1^−/−^ cells were observed. Furthermore, the loss of YME1L increased mitochondrial ROMO1 levels drastically, suggesting ROMO1 is a degradation substrate of YME1L (Figure 1), thus YME1L can mediate its own import via a self-regulatory mechanism [25]. ROMO1 displays high sequence similarity to yeast MIM protein Mgr2, which was suggested to perform multiple functions at the TIM23 complex [26,27]. Recent structural and biochemical studies show that the yeast TIM23 complex contains a heterotrimer of the subunits Tim23, Tim17, and Mgr2 [28,29]. During substrate translocation, Mgr2 seals the lateral opening of the Tim17 cavity to facilitate the translocation process of substrate polypeptides. Although the mitochondrial protein import machineries of yeast and human counterparts are highly conserved, there are differences in the composition and molecular function of subunits [21]. It would be interesting to understand whether Mgr2 is required for Yme1 import and if itself a degradation substrate of Yme1 in yeast in the future. Taken together, these studies show that there is an interplay between Yme1/YME1L and TIM23 complex in mitochondrial protein import and homeostasis.

**Figure 2.**
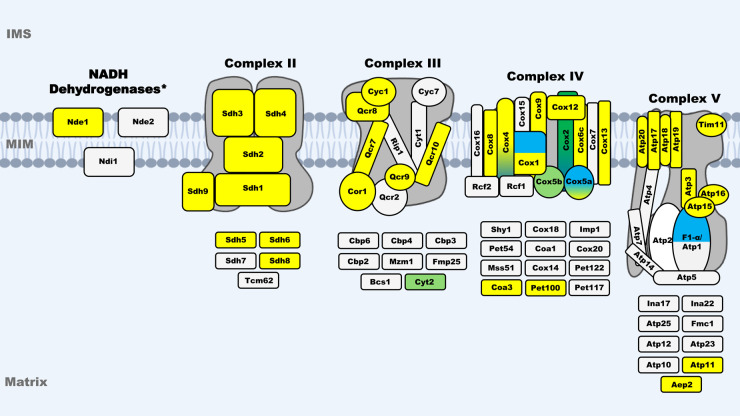
Schematic diagram showing the relationships between i-AAA and OXPHOS complex subunits. Based on yeast *S. cerevisiae*, the complex subunits are shown at their rough positions within their respective complexes, and the assembly factors are shown below each complex. The colour representations: Dark green for Yme1 confirmed substrates; light green for putative substrates of Yme1; yellow for proteins with decreased abundances in *Δyme1* yeast cells; grey for proteins with no obvious abundance changes in *Δyme1* yeast mitochondria. In addition, dark blue is used to present confirmed substrates of human YME1L, and light blue for putative substrates of YME1L and AFG3L2. The subunit NDUFB6 of Complex I is a putative substrate of YME1L.

Known substrates of Yme1 also include the highly conserved soluble small Tim proteins of the IMS, which play important roles during the import of hydrophobic membrane proteins including Tim23 [30,31]. Tim9 and Tim10 assemble into an essential heterohexameric Tim9/10 complex, which chaperones newly imported hydrophobic preproteins from the TOM complex to TIM22 translocase for membrane insertion (Figure 2). Whilst the small Tim proteins are imported through a redox-sensitive MIA40 pathway [32,33], misfolded and unassembled small Tim proteins are targeted for proteolytic degradation within mitochondria by Yme1 [15,34,35]. Furthermore, Spiller et al. [15] showed that unassembled Tim10 was more susceptible to degradation by Yme1 *in vitro* than Tim9, and the complex formation protects them from degradation by Yme1. Tim8 and Tim13 also form a heterohexameric chaperone complex in the IMS, they are required for the import of Tim23 via the TIM22 complex when the membrane potential is low [36,37]. Tim12 is the only small Tim located peripherally on the MIM, as an essential component of the TIM22 translocase acting downstream of the Tim9/10 complex [38]. Moreover, a recent study identified cross-interactions between the TIM22 translocase complex and Yme1 [39]. The study demonstrated that disrupting the TIM22 complex in yeast, either by subunit deletion or mutation, would rescue the growth defects of *YME1* deletion mutants under respiratory conditions [39]. It also showed that Yme1 and TIM22 complex are in proximity to each other within the MIM, and loss of Yme1 would reduce the stability of the TIM22 complex and its protein import efficiency, especially under heat stress [39].

Collectively, these studies suggest that Yme1 plays an important role in mitochondrial protein homeostasis by regulating the function and efficiency of the TIM22 and TIM23 import machineries. Moreover, Yme1 was shown to mediate the degradation of MOM proteins Tom22 and Om45 [17] and serves as a nonconventional translocation motor during the import of polynucleotide phosphorylase into the mitochondrial IMS [40]. Such a protein import regulatory mechanism may prove more efficient in maintaining MIM protein homeostasis than direct substrate degradation. It may also provide a reason why only a few degradation substrates of Yme1 were experimentally confirmed, whilst the abundances of many mitochondrial proteins are increased in the *Δyme1* mitochondria.

### Role of Yme1 in the homeostasis of OXPHOS complex proteins

It is widely accepted that Yme1 plays an important role in OXPHOS complex functions due to the identification of putative substrates including Cox2, Qcr2, Atp4, Atp6, Atp17, and impacts of *YME1* deletion on mitochondria function, such as impaired respiration with abnormal globular mitochondria. Early investigations identified Complex IV subunit Cox2 as a Yme1 degradation substrate [16,41–43]. In particular, Weber et al. [16] had observed the accumulation of unassembled Cox2 in *YME1* deletion yeast and strains with functionally impaired Yme1 mutants. These studies showed Yme1-mediated Cox2 degradation in an ATP and divalent metal ion-dependent manner. A mass spectrometry-based study on protein aggregates in the mitochondria isolated from *YME1* deletion (*Δyme1*) yeast had identified Cox2, among other ETC components and assembly factors including Cyc1, Rcf2, and Cbp4, accumulated in a protein aggregation fraction [44]. This further supports the view that Yme1 is responsible for Cox2 degradation.

Furthermore, a study on ETC complex regulation and degradation under different stresses demonstrated increased turnover of NADH:ubiquinone oxidoreductases Ndi1 and Nde1 (yeast equivalent to Complex I) and Complex III components [45]. A recent study identified that Nde1 forms two distinct topomers in mitochondria, one residing in the IMS while the other spans the MOM and is exposed to the cytosol, and degradation of the latter is regulated by the cytosolic proteasome system and mitochondrial Yme1 [46]. The degradation of Qcr2, a subunit of Complex III, was significantly decreased in *YME1* deletion background upon active respiratory [45], suggesting Qcr2 is a degradation substrate of Yme1 under active respiratory growth. In a study on the relationship between Yme1, ETC complex assembly factor Oxa1 Lemaire et al. [47] showed that while F_0_ subunits Atp4, Atp6, and Atp17 levels were dramatically reduced in *Δoxa1* strain, but were restored in the double deletion mutant *Δoxa1Δyme1* suggesting Yme1 may be responsible for the degradation of these F_0_ subunits. Interestingly, this study also showed degradation of Complex IV subunits, including Cox2, in the *Δoxa1Δyme1* double mutant [47], indicating that Yme1 is not the only proteinase responsible for the degradation of these subunits.

Whilst most studies on this topic are performed using yeast as a model, few studies focused on human YME1L. A study demonstrated that unassembled subunits of Complex IV (Cox2 and Cox4) were degraded by YME1L [48]. Moreover, a later study demonstrated YME1L and AFG3L2 (Afg3 in yeast, subunit of m-AAA) double knock-down not only resulted in the accumulation of the subunits of Complex I (NDUFB6), IV (COX1, COX5A, COX4), and V (F1-alpha), but also impairment of the activities of these complexes [49]. These results highlight the key role YME1L plays in regulating ETC complex in human. Given the similarity in sequence identity between yeast and human (YME1L) homologues [50], it is possible these homologues also have similar interaction partners and degradation substrates, hence sharing a similar role in maintaining mitochondrial and OXPHOS function.

Recently, the effect of *YME1* deletion on subunits of OXPHOS complexes was studied in a systematic manner [19]. In this study, mitochondrial proteomics analysis revealed that the abundances of several subunit proteins of OXPHS. They include five components of Complex II (Sdh1–4, Sdh9), seven subunits of Complex III (Cyc1, Cor1, Qcr2, and Qcr7–10), eight of Complex IV (Cox1, Cox4, Cox5A, Cox6c, Cox8, Cox9, Cox12, and Cox13), and seven of Complex V (Atp3, Atp11, Atp15, Atp16, Atp17, Atp19, and Atp20), were decreased in the *Δyme1* mitochondria (Figure 2). Thus, they are unlikely to be degradation substrates of Yme1. Meanwhile, the abundance of Cox5b (subunit of Complex IV) and assembly factors Cyt2 of Complex III and Aep2 of Complex V were statistically increased >1.5-fold. The abundance of Cox2 was increased by 1.3-fold, albeit statistically not significant (*p* > 0.05), which is consistent with the previous finding that *YME1* deletion leads to the accumulation of unassembled Cox2 [16]. In addition, complex activity assays in conjunction with immunoblotting suggested that the decreased activities of the ETC complexes were resulted from the decreased protein abundance of complex subunits, rather than the rate of the complex assembly [19]. A possible explanation for this observation is that *YME1* deletion enhanced the activity of m-AAA which may be able to degrade those subunits of OXPHOS. However, proteomic studies showed no changes in the abundance of m-AAA subunits in *YME1* deletion backgrounds [19,51,52].

Collectively, despite these studies, the function of Yme1 on OXPHOS complex constituents remains elusive as seemingly complex and contrasting data were reported in the limited studies. These results indicate that the role of Yme1 in OXPHOS is more complicated, its main function here may not act as a proteinase for the degradation of misfolded or unassembled OXPHOS subunits directly. Other mechanisms, such as by regulating the import efficiency of the subunit proteins via a currently unknown process, are possible. Detailed molecular and cellular studies are required to address the role of Yme1 in maintaining OXPHOS activity.

### Functional interplay between Yme1/YME1L, mitochondrial lipid biosynthesis and signalling

**Figure 3.**
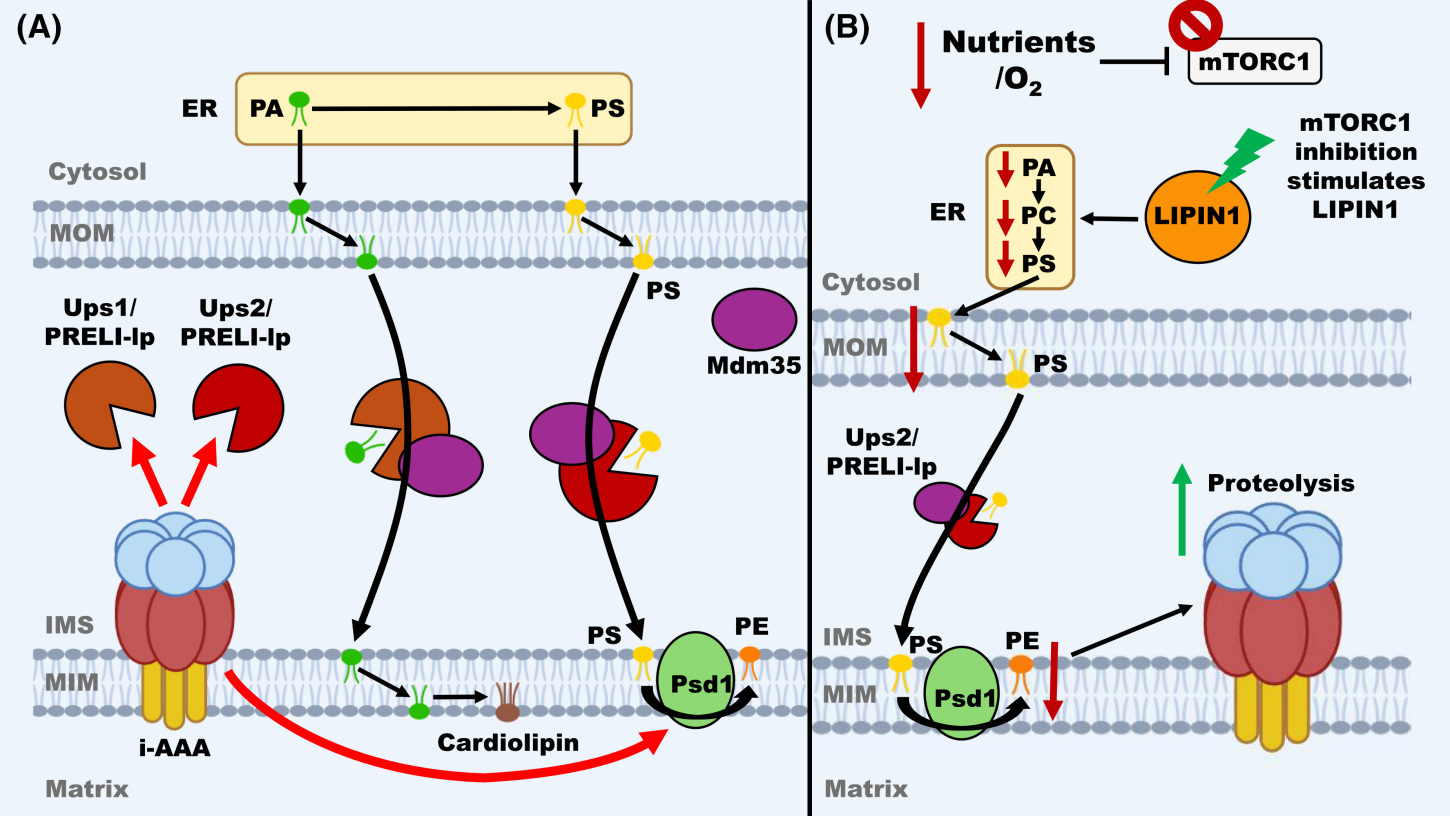
Schematic diagram for the interplay between i-AAA proteinase and mitochondrial lipid metabolism and signalling. (**A**). Cardiolipin and PE (phosphatidylethanolamine) synthesis. i-AAA plays a role in regulating cardiolipin and PE synthesis by degradation of the Ups/PRELI-lp lipid transporters and Psd1 (phosphatidylserine decarboxylase 1) as indicated by thick red arrows. Mdm35 complex with Ups/PRELI-lp can prevent their degradation by i-AAA. (**B**). mTORC1-dependent PE synthesis and increased i-AAA proteolysis. mTORC1 inhibition upon nutrient or oxygen deprivation activates LIPIN1 and consequently results in decreased level of PE in the MIM, which in turn increases mammalian i-AAA YME1L proteinase activity.

Specific phospholipid composition in mitochondria is essential for mitochondrial activities. Whilst most phospholipids are synthesised in the endoplasmic reticulum (ER) and transported to mitochondria, cardiolipin, and phosphatidylethanolamine (PE) are produced in mitochondria [53]. In yeast, as shown in Figure 3A, several proteins are involved in these lipid biosynthesis pathways, including Ups1, Ups2, and Psd1, which were identified as being substrates of Yme1 [14,54,55]. Cardiolipin is a critical component of the MIM, responsible for maintaining its mechanical stability and electrochemical potential, and is required for the integrity of several protein complexes in the MIM, including the TIM23 translocase [14,55,56]. As shown in Figure 3A, cardiolipin is synthesised from phosphatidic acid (PA) imported from the ER [57]. Ups1 plays an important role in the intra-mitochondrial trafficking of PA from the MOM to the MIM and thus cardiolipin synthesis [14,55]. PE like cardiolipin is also a critical component of mitochondrial membranes, where its conical shape assists in forming topological structures such as cristae and aids in membrane protein folding [58]. PE is synthesised from phosphatidylserine (PS) imported from the ER, where it is then trafficked from the MOM to the MIM by Ups2 [54,55]. Furthermore, the synthesis of PE is mediated in part by phosphatidylserine decarboxylase 1 (Psd1) and mitochondrial contact site and cristae organising system (MICOS) [54,59]. While Yme1 was demonstrated to aid in the degradation of Pds1; however, it is unclear if this is due to its proteolytic activity [54]. In addition, it was reported that Ups1 and Ups2 have antagonistic effects on cardiolipin levels in mitochondria [56]. Interestingly, they also affect the assembly state of TIM23 and its association with PAM, hence affecting mitochondrial protein import efficiency in an opposing manner [56].

Whilst solitary Ups1 and Ups2 are unstable due to proteolysis, Mdm35 (mitochondrial distribution and morphology protein 35) can interact with both proteins and protect them against proteolysis [14,55]. Therefore, Mdm35 and Yme1 share a regulatory partnership regulating Ups1 and Ups2 activities by the dynamic association and dissociation of Mdm35 with either protein. However, the overall regulatory impact of Yme1 on cardiolipin synthesis is yet to be fully understood. For example, a recent study found no noticeable differences in the cardiolipin content and membrane potential observed in *Δyme1* mitochondria, despite the abundances of both Ups1/2 increasing [19]. Potentially this may be due to the opposing effects of Ups1/2 in cardiolipin metabolism compensating each other, although this antagonism is still yet to be defined. Hence, it will likely take more research to understand the exact roles of Yme1/YME1L in the cardiolipin biosynthesis.

In mammals, these cardiolipin and PE synthesis pathways are conserved, with PRELI-lps (protein of relevant evolutionary and lymphoid interest-like proteins) acting as homologues of Ups1/2, and TRIAP1 as a functional homologue of Mdm35 [60]. Additionally, a similar functional relationship between YME1L and PRELI-lp/TRIAP1 was shown [60], indicating that these regulatory systems are highly conserved. Regarding PE synthesis, there is little evidence to suggest that YME1L has any regulatory influence on PISD, the mammalian functional homologue of Psd1. However, Ups2 and PRELI-lps are involved in the transport of PS and regulated by Yme1/YME1L, which could indicate a much more complex relationship between lipid metabolism and proteolytic activity in either model.

A recent study showed that YME1L regulates the rewiring of the mitochondrial proteome in response to hypoxia or nutrient starvation via a lipid signalling pathway [24]. It showed that upon inhibition of mTORC1 (mammalian target of rapamycin 1), a lipid signalling cascade was induced via LIPIN1 (a PA phosphatase). Consequently, the PE level in the MIM is decreased, which promotes proteolysis by YME1L and rewiring of the mitochondrial proteome (Figure 3B) [24]. mTOR is a phosphatidylinositol 3-kinase-related kinase that forms mTOR Complexes 1 and 2 (mTORC1 and 2) [61,62]. mTORC1 co-ordinates signals from growth factors and metabolism monitoring pathways to up-regulate cell-growth activities or to promote catabolic cell pathways in the face of nutrient deprivation while mTORC2 governs cell proliferation and cell survival mechanisms [61]. This recent study found that the inhibition of mTORC1 activates a lipid-dependent signalling cascade mediated by the activation of a PA phosphatase called LIPIN1 [24]. Herein, the activation of LIPIN1 depletes PA levels, which in turn reduces the synthesis of phosphatidylcholine (PC), PS, and ultimately PE level, which consequently led to up-regulation of YME1L proteolysis activity [20,24]. It is surmised that the up-regulation of YME1L activity inhibits mitochondrial protein import [24]. Additionally, given that PRELI-lps (importing PS for PE synthesis) are substrates of YME1L, it appears that this mTORC1-LIPIN1-YME1L axis may mediate a positive feedback loop resulting in further reductions of PE levels and heightened YME1L proteolysis [20]. Additionally, the paper identified that hypoxia-inducing factor α (HIF-α) can activate this mTORC1–LIPIN1–YME1L axis (Figure 3B), which was associated with the development of pancreatic ductal adenocarcinoma (PDAC) both *in vitro* and in murine xenografts [24]. This conserved pathway found in yeast with complexes TORC1 and TORC2 as constituents [61]. Therefore, it would be reasonable to hypothesise that a similar degree of interaction exists between TOR and Yme1 as does mTOR and YME1L, and will be interesting to see if future research can identify a conserved axis in both models.

## The role of i-AAA protease in modulating mitochondrial dynamics

Apart from the important roles in mitochondrial protein quality control, Yme1/YME1L also plays a crucial role in modulating mitochondrial dynamics via regulating mitochondrial fusion and fission. These processes are crucial for maintaining mitochondrial function, quality control, and overall cellular health. Since the role of Yme1/YME1L on the regulation of mitochondrial dynamics has been well discussed in a recent review [20], and no further progress has been made since, in this mini review, we discuss this topic briefly. In human, the dynamin-like GTPase OPA1 (Mgm1 in yeast) regulates mitochondrial fusion at MIM, inactivation of OPA1 results in mitochondrial fragmentation and decreased activities [63,64]. Two forms of OPA1 were identified, the MIM anchored long form (L-OPA1) and the soluble short form (S-OPA1) after proteolytic cleavage of the N-terminal anchor [63]. The maintenance of mitochondrial morphology depends on a good balance between L-OPA1 and S-OPA1. Studies have shown that while L-OPA1 mediates mitochondrial fusion S-OPA1 promotes mitochondrial fragmentation [65]. Two proteases, OMA1 and YME1L, were found to proteolyticly process L-OPA1 at two separate but neighbouring cleavage sites to creating two S-OPA1 isoforms. Whilst OMA1 cleaves OPA1 at S1, YME1L cleavage occurs at S2 [66,67]; but the functional difference between the two S-OPA1 is unknown. However, studies had shown that YME1L-mediated processing of OPA1 increased during respiratory growth, while OMA1L-mediated OPA1 processing was elevated when under various cellular stresses [20]. Moreover, YME1L is also responsible for the complete degradation of OPA1 [24]. Therefore, YME1L has an additional level of regulatory effect on OPA1, which indirectly regulates downstream pathways in mitochondrial dynamics, cristae morphogenesis, and respiration to altered metabolic demands.

In yeast, the homologue Mgm1 also exists as long and short forms as product of proteolytic processing, acting in a similar manner to the human homologue [65]. However, it is processed by a rhomboid protease Pcp1, rather than Oma1 and Yme1 [68]. Further research is required to elucidate whether Yme1 interacts with mitochondrial fusion machinery in yeast.

## Perspectives

- Yme1 plays an important in the regulation of a wide range of mitochondrial functions, from mitochondrial protein biogenesis, energy production to organelle dynamics, and lipid signalling.
- Further systematic studies at detailed molecular and cellular levels would help elucidate the mechanism by which Yme1 acts on these proteins and the broader effect on mitochondrial proteostasis and function.
- Mammalian studies showed that YME1L mutations lead to neurological and developmental disorders due to mitochondrial dysfunction and fragmentation [69–71]. Further investigation into the role of i-AAA in mitochondrial quality control under the context of pathogenesis may provide insight into developing new treatments and therapeutics.

## Open Access

Open access for this article was enabled by the participation of the University of Manchester in an all-inclusive *Read & Publish* agreement with Portland Press and the Biochemical Society under a transformative agreement with JISC.

## Abbreviations

ER: endoplasmic reticulum
IMS: intermembrane space
MIM: mitochondrial inner membrane
MOM: mitochondrial outer membrane
mTORC1: mammalian target of rapamycin 1
OXPHOS: oxidative phosphorylation
PA: phosphatidic acid
PE: phosphatidylethanolamine
PS: phosphatidylserine
Psd1: phosphatidylserine decarboxylase 1
